# Impact of the Presence of Medical Equipment in Images on Viewers’ Perceptions of the Trustworthiness of an Individual On-Screen

**DOI:** 10.2196/jmir.1986

**Published:** 2012-07-10

**Authors:** Moyez Jiwa, Stephan Millett, Xingqiong Meng, Vivien M Hewitt

**Affiliations:** ^1^Curtin Health Innovation Research InstituteCurtin UniversityPerth, Western AustraliaAustralia; ^2^Research & DevelopmentAdministration & FinanceCurtin UniversityPerth, Western AustraliaAustralia; ^3^School of Public HealthCurtin UniversityPerth, Western AustraliaAustralia; ^4^Fremantle HospitalFremantle, Western AustraliaAustralia

**Keywords:** Icons, semiotics, stethoscope, doctors, trustworthiness

## Abstract

**Background:**

It is now common practice for doctors to consult patients by means other than face-to-face, often appearing before the patient on a computer screen. Also, many websites are using depictions of health professionals to increase the credibility of their services. Being trustworthy is an essential attribute for successful ehealth services. Little is known about which depicted accessories make a health professional appear more trustworthy.

**Objective:**

To estimate the odds of an individual on-screen being rated trustworthy when viewed in a static image holding or wearing specific items of medical equipment.

**Methods:**

We surveyed consecutive people attending community pharmacies to collect prescriptions in Western Australia. Respondents were presented with a series of 10 photographs, generated at random, of a man with varying numbers and combinations of medical equipment: stethoscope, reflex hammer, surgical scrubs, otoscope, and pen. They were then invited to rate the man as honest, trustworthy, honorable, moral, ethical, or genuine, or a combination of these, on the Source Credibility Scale.

**Results:**

A total of 168 of 250 people gave informed consent, for a participation rate of 67.2%. There were 102 female and 66 male respondents. Of the 168 respondents, 96 (57%) were born in Australia and 102 (60.7%) were attending medical practices with more than one general practitioner. The mean age of respondents was 47 (SD 16) years (range 26–92 years). When only 1 item was present in an image, the stethoscope was associated with the highest odds for the person being considered honest (odds ratio [OR] 2.6, 95% confidence interval [CI] 1.6–4.3), trustworthy (OR 2.3, 95% CI 1.4–3.8), honorable (OR 2.7, 95% CI 1.6–4.5), moral (OR 2.4 95% CI 1.4–4.1), ethical (OR 2.6, 95% CI 1.5–4.6), and genuine (OR 1.8, 95% CI 1.0–3.1). The presence of a stethoscope increased the odds of the person being rated in a positive light in all photographs in which it was included.

**Conclusions:**

When an individual is portrayed in a static image, concurrent presentation of 3 or more items of medical equipment, and especially a stethoscope, is likely to exert a positive influence on the viewers’ perceptions of the qualities of the person depicted.

## Introduction

When people are concerned about their physical or psychological well-being, they may consult a doctor. When a patient consults a doctor face-to-face, all five senses affect the experience: sight, hearing, touch, smell (eg, medications, disinfectants, and tissues), and sometimes taste (eg, medications, and equipment used to examine the mouth or throat) [1-5]. In consultations where the doctor and patient are not even in the same room, there are limits to the extent to which all the senses can be engaged [6]. It is important that a doctor (or online ehealth service or website) be perceived as trustworthy, especially in circumstances in which there are limits to the engagement of the senses as occurs, for example, when people interact with doctors via a computer or when a doctor is portrayed in a static image in support of a message on a website.

When someone is experiencing symptoms, the condition does not require the presence of objective organic pathological analysis. Indeed, some people experience relief from symptoms despite being treated with a placebo or inactive drug [7,8]. It has been demonstrated in conditions that are not the result of organic pathology that a doctor has powers to relieve disease in a manner analogous to that of a placebo [9]. In common with many placebos, the physical appearance of the doctor may account for some of the therapeutic response. As McCroskey stated, “No message is received independently of its source or presumed source” [10]. Various writers have agreed that there is a dimension to the perception of an individual that can be referenced as trustworthiness (character, sagacity, safety, and honesty) [11].

The literature records many studies in which credibility and persuasive communication have been tested with specific reference to the physical appearance of doctors. In an experiment conducted by computer scientists, advice was shown to be much more persuasive when presented as coming from doctors as depicted in static pictures [12]. Similarly, previously published studies have reported that patients prefer traditional physicians’ attire consisting of a white coat and professional dress [13]; other studies indicate that patients are equally satisfied with their physicians regardless of casual or business attire [14]. In a more recent experiment, researchers have concluded that doctors working with a primarily older population may find their patients prefer their physicians to wear white coats, whereas a large majority of parents do not expect their child's doctor to wear a white coat and that the a parent’s trust is not compromised by less-traditional physicians’ attire [15].

In addition to what the doctor is wearing, we must also consider what he or she is seen to be holding, or using. For example, the stethoscope has been the cornerstone of medical diagnostics for nearly 200 years. This monaural device improved physicians’ ability to hear clues to their patients’ underlying pathology and thus apply the appropriate treatment in many cases. It has been eulogized by many commentators, such as the following:

*I contend that the stethoscope best symbolizes the practice of medicine. Whether absentmindedly worn around the neck like an amulet or coiled gunslinger-style in the pocket, ever ready for the quick draw, the stethoscope is much more than a tool that allows us to eavesdrop on the workings of the body. Indeed, it embodies the essence of doctoring: using science and technology in concert with the human skill of listening to determine what ails a patient. *[16]

The stethoscope is one of several medical instruments that are recognized by laypeople as belonging in a doctor’s office and that have significance in the consultation that extends beyond the instrument’s physical functionality. Similarly, reflex hammers and other items used in clinical examination have developed an iconic status for doctors [17]. In this study we hypothesized that in the absence of any other information, an individual appearing in a static image may be rated as more trustworthy when he is viewed holding or wearing specific items of medical equipment. Further, we hypothesized that some items of medical equipment would have a greater influence than others on the perception of trustworthiness.

## Methods

We obtained human research ethics approval from Curtin University (approval number RD-23-10) before commencing the study. All respondents provided consent before data collection. Consecutive people waiting in six community pharmacies to collect prescriptions for drugs prescribed by a doctor were presented with a series of photographs on an iPad. Each photograph presented an image of a man with a neutral facial expression who was wearing a casual shirt. Some of the images contained medical equipment. The respondents were not told anything about the person in the photograph. The photographs were presented as a series of 10 consecutive images containing an increasing number of items of medical equipment ranging from 0 to 5. Each series of 10 was unique to each participant and drawn at random from a pool of images. The equipment (collectively called icons here) consisted of a stethoscope, otoscope, reflex hammer, surgical scrubs, and pen. We chose these icons from the list of items that have been recommended as necessary in a general practitioner’s bag [18]. The icons were presented in increasing numbers and in the following order, with the collection of icons chosen at random: (1) no icons (1 photo, shown first), (2) 1 icon (2 photos, each containing 1 icon selected at random), (3) 2 icons (2 photos, each containing 2 icons selected at random), (4) 3 icons (2 photos, each containing 3 icons selected at random), (5) 4 icons (2 photos, each containing 4 icons selected at random), and (6) 5 icons (1 photo, shown last).

**Figure 1:**
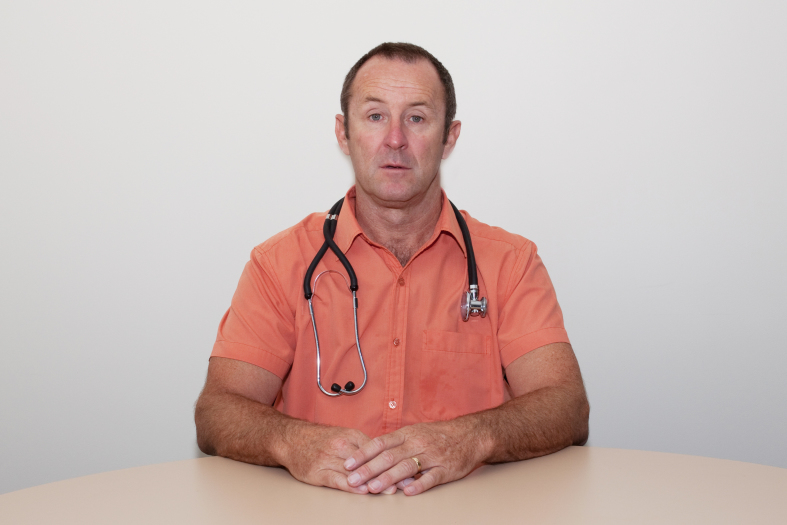
Example of an image shown to the respondents.

Figure 1 and Multimedia Appendix 1, Multimedia Appendix 2, Multimedia Appendix 3, Multimedia Appendix 4, Multimedia Appendix 5, and Multimedia Appendix 6 illustrate examples of the photos.

**Figure 2:**
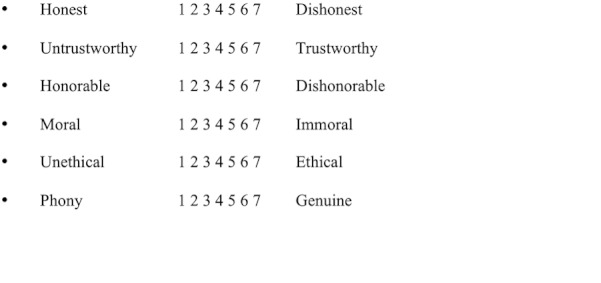
Elements of the Source Credibility Scale.

The participants were invited to rate the individual using the trustworthiness measure of the Source Credibility Scale (SCS) [11]. The SCS is an 18-item survey comprising three separate measures that capture respondents’ perceptions of an individual’s “competence, trustworthiness, and goodwill/caring.” The three measures in the SCS each represent a unique construct, the scores for which must be considered in isolation from the others. Each individual measure may validly be subject to regression analysis [11]. For this study and to keep the required sample size to a minimum, we invited the participants to respond only to the six questions from the SCS that make up the trustworthiness measure [11]. The internal consistency (alpha reliability) of the trustworthiness measure is .93. Each element was scored from 1 to 7 at either end of the scale, as shown in Figure 2.

### Sample Size

For logistic regression, to estimate the odds of a specific outcome within 80% confidence intervals at 5% significance, in this case scoring an image as trustworthy and modeling 11 explanatory variables, we required 138 respondents based on a moderate effect size [11,19].

### Statistical Analysis

We used ordinal logistic regression to examine the influence on primary outcomes of icons presented in the photos. The influence was calculated as the odds of a person being rated honest, trustworthy, honorable, moral, ethical, or genuine, or a combination of these, after adjusting the respondents’ characteristics (eg, sex, age group, and country of birth). Five predictor variables (the icons, ie, the stethoscope, otoscope, reflex hammer, surgical scrubs, and pen) were assessed after controlling for respondents’ demographics and experience of consulting doctors. This consulting experience included the type of medical practice frequented (solo practitioner or group practice), number of general practitioner visits, and number of hospital visits.

Regression analysis was performed to assess the effect of the 5 icons and the different combinations of icons on rating for honest, trustworthy, etc. A variable that defined the 5 icons (model 1), the different combination of icons (2 icons, 3 icons, 4 icons: models 2–4), and all 5 icons (model 5) were used in the corresponding models. In each model, the group of vignettes (photographs) with no icon was treated as the reference group, and the groups of vignettes with 1, 2, 3, 4, or 5 icons were compared with the reference group respectively in the regression model. The lack of independence between individual participants, which causes intragroup correlation, was adjusted in the models through estimating the clustered robust standard errors. Statistical analysis was performed using Stata Statistical Software (IC 11.1; State Corp LP, College Station, TX, USA).

## Results

We invited 250 people to participate, and 168 completed the survey, giving a participation rate of 67.2%. Demographic details are presented in Table 1. There were more female than male respondents, and more respondents attending medical practices with more than one general practitioner. Most respondents were born in Australia, and the mean age of respondents was 47 (SD 16) years (range 26–92 years).

**Table 1:** Demographic characteristics of respondents (n = 168).

Characteristic		n	%	*P *value^a^
**Age (years)**				.64
	≤45	81	48%	
	>45	87	52%	
**Sex**				.006
	Male	66	39%	
	Female	102	60.7%	
**Country of birth**				.06
	Australia	96	57%	
	Other	72	43%	
**Type of medical practice frequented**				.006
	Clinic with more than one general practitioner	102	60.7%	
	Solo practitioner	66	39%	

^a^ *P *values were derived from 2-sample test of proportion.

Table 2, Table 3, and Table 4 show that the stethoscope was more likely to be present in images where the person in the photograph was regarded as one or more of honest, trustworthy, honorable, moral, ethical, or genuine. This was particularly true in images that contained only one piece of equipment. However, the stethoscope was also more likely to be present in images credited with any of these characteristics when multiple pieces of equipment were presented.

**Table 2:** Data for person in photograph being perceived as honest and trustworthy when 1 or more icons^a ^are present versus no icons.

Model		Honest		Trustworthy	
		OR^b^	95% CI^c^	OR	95% CI
**1(1 icon)**					
	S	2.6	1.6–4.3***	2.3	1.4–3.8***
	O	1.6	1.1–2.4*	1.4	0.9–2.3
	RH	1.5	0.9–2.5	1.6	1.0–2.5*
	SS	1.3	0.8–2.1	1.2	0.8–1.9
	*P*	1.3	0.8–2.1	1.2	0.8–2.0
	No icon^d^	1.0		1.0	
**2 (2 icons)**					
	S+RH	3.9	2.1–7.4***	2.3	1.1–4.6*
	S+SS	2.5	1.5–4.3***	1.3	0.7–2.5
	S+P	2.5	1.3–4.8**	2.7	1.5–4.8***
	O+RH	2.9	1.7–4.9***	1.8	0.9–3.4
	O+P	1.8	0.8–3.7	2.3	1.2–4.3*
	RH+SS	2.5	1.3–4.8**	1.4	0.6–3.1
	RH+P	1.8	0.9–3.4	1.8	1.0–3.2^e^
	SS+P	1.7	0.9–3.4	1.9	1.0–3.5^e^
	O+P	2.4	1.0–5.5*	4.0	2.3–7.0***
	No icon^d^	1.0		1.0	
**3 (3 icons)**					
	S+O+RH	3.0	1.6–5.7***	3.1	1.6–5.8***
	S+O+RH	4.0	2.3–6.8***	2.3	1.3–4.2**
	S+O+P	4.0	2.1–7.6***	3.9	2.0–7.5***
	S+RH+SS	2.6	1.3–5.1**	2.8	1.5–5.2***
	S+RH+P	2.3	1.0–5.1*	2.2	1.1–4.5*
	S+SS+P	3.4	1.8–6.4***	2.7	1.4–5.4**
	O+SS+P	1.8	0.7–4.5	1.7	0.5–5.3
	No icon^d^	1.0		1.0	
**4 (4 icons)**					
	S+O+RH+SS	4.4	2.5–7.7***	2.6	1.4–4.9**
	S+O+RH +P	4.0	2.5–6.4***	3.0	1.9–4.8***
	S+O+SS+P	3.7	2.2–6.1***	3.6	2.3–5.7***
	S+RH+SS+P	2.1	1.3–3.4**	2.8	1.8–4.6***
	O+RH+SS+P	3.4	1.9–6.2***	1.6	0.8–3.2
	No icon^d^	1.0		1.0	
**5 (5 icons)**		3.7	2.4–5.5***	3.7	2.5–5.5***
	No icon^d^	1.0		1.0	

^a ^Icons are as follows: S = stethoscope, O = otoscope, RH = reflex hammer, SS = surgical scrubs,*P* = pen.

^b ^Odds ratio.

^c ^Confidence interval.

^d ^Photo with no icon group was the reference group for each of the models.

^e ^95% CI was rounded to 1.00 but the *P *value was >.05.

*P < .05, **P < .01, ***P < .001.

**Table 3:** Data for person in photograph being perceived as honorable and moral when 1 or more icons^a ^are present versus no icons.

Model		Honorable		Moral	
		OR^b^	95% CI^c^	OR	95% CI
**1(1 icon)**					
	S	2.7	1.6–4.5	2.4	1.4–4.1**
	O	1.3	0.8–2.1	1.3	0.8–2.2
	RH	1.6	1.0–2.6^e^	1.7	1.1–2.6*
	SS	1.3	0.8–2.2	1.4	0.9–2.3
	*P*	1.7	1.0–2.6*	1.1	0.7–1.8
	No icon^d^	1.0		1.0	
**2 (2 icons)**					
	S+RH	2.2	1.1–4.5*	2.1	1.1–4.1*
	S+SS	2.5	1.5–4.2***	2.0	1.2–3.4**
	S+P	1.7	0.9–3.3	2.2	1.3–4.0**
	O+RH	2.4	1.3–4.5**	2.4	1.4–4.3**
	O+P	1.9	0.9–3.7	1.5	0.7–3.5
	RH+SS	2.3	1.1–4.7*	2.0	1.0–4.0^e^
	RH+P	2.7	1.3–5.6**	2.7	1.4–5.0**
	SS+P	2.7	1.4–5.1**	2.5	1.4–4.5**
	O+P	1.7	0.7–4.0	1.9	0.8–4.3
	No icon^d^	1.0		1.0	
**3 (3 icons)**					
	S+O+RH	3.5	1.9–6.6***	3.3	1.8–6.1***
	S+O+SS	3.0	1.7–5.5***	2.9	1.6–5.1***
	S+O+P	2.4	1.2–5.1*	2.3	1.1–5.1*
	S+RH+SS	3.0	1.5–6.0**	2.5	1.3–5.0**
	S+RH+P	2.4	1.1–5.1*	2.1	1.0–4.3*
	S+SS+P	4.2	2.3–7.6***	3.9	2.2–7.1***
	O+SS+P	2.3	1.1–5.1*	2.0	1.0–4.0^e^
	No icon^d^	1.0		1.0	
**4 (4 icons)**					
	S+O+RH+SS	3.7	2.0–6.9***	3.6	2.0–6.5***
	S+O+RH+P	3.3	2.0–5.5***	3.0	1.9–4.8***
	S+O+SS+P	2.6	1.6–4.3***	2.5	1.5–4.2***
	S+RH+SS+P	2.4	1.4–3.9***	1.8	1.1–3.1*
	O+RH+SS+P	3.6	2.0–6.4***	3.4	2.1–5.7***
	No icon^d^	1.0		1.0	
**5 (5 icons)**		3.1	2.1–4.6***	3.1	2.2–4.4***
	No icon^d^	1.0		1.0	

^a ^Icons are as follows: S = stethoscope, O = otoscope, RH = reflex hammer, SS = surgical scrubs,*P* = pen.

^b ^Odds ratio.

^c ^Confidence interval.

^d ^Photo with no icon group was the reference group for each of the models.

^e ^95% CI was rounded to 1.00 but the *P *value was >.05.

*P < .05, **P < .01, ***P < .001.

**Table 4:** Data for person in photograph being perceived as ethical and genuine when 1 or more icons^a ^are present versus no icons.

Model		Ethical		Genuine	
		OR^b^	95% CI^c^	OR	95% CI
**1(1 icon)**					
	S	2.6	1.5–4.6***	1.8	1.0–3.1*
	O	1.7	1.0–2.8^e^	1.4	0.9–2.3
	RH	1.5	0.9–2.6	1.7	1.1–2.6*
	SS	1.2	0.7–2.1	1.3	0.8–2.0
	*P*	1.3	0.8–2.1	0.9	0.5–1.5
	No icon^d^	1.0		1.0	
**2 (2 icons)**					
	S+RH	2.8	1.4–5.7**	2.1	1.1–4.0*
	S+SS	1.6	0.9–3.1	1.3	0.7–2.4
	S+P	2.8	1.5–5.1***	2.5	1.4–4.1***
	O+RH	2.1	1.1–4.3*	1.5	0.7–3.2
	O+P	2.8	1.4–5.5**	2.3	1.2–4.3*
	RH+SS	2.9	1.4–5.7**	2.7	1.4–5.2**
	RH+P	2.6	1.4–4.5***	1.8	1.0–3.2*
	SS+P	2.8	1.4–5.5**	2.0	1.0–3.9^e^
	O+P	3.7	2.1–6.7***	3.5	1.6–7.6***
	No icon^d^	1.0		1.0	
**3 (3 icons)**					
	S+O+RH	3.3	1.8–5.9***	2.4	1.3–4.1**
	S+O+SS	2.6	1.4–5.0**	2.1	1.1–3.8*
	S+O+P	3.4	1.8–6.4***	3.1	1.6–5.7***
	S+RH+SS	3.2	1.6–6.3***	2.9	1.6–5.3***
	S+RH+P	2.1	0.9–4.6	1.7	0.7–4.1
	S+SS+P	3.4	1.8–6.7***	2.5	1.2–5.4*
	O+SS+P	2.3	1.0–5.4^e^	1.5	0.7–3.3
	No icon^d^	1.0		1.0	
**4 (4 icons)**					
	S+O+RH+SS	3.5	2.0–6.2***	2.9	1.6–5.1***
	S+O+RH+P	2.9	1.7–5.0***	2.6	1.6–4.3***
	S+O+SS+P	3.1	1.9–5.0***	2.6	1.6–4.3***
	S+RH+SS+P	2.9	1.8–4.8***	2.5	1.6–3.9***
	O+RH+SS+P	2.4	1.3–4.5**	1.8	1.0–3.5^e^
	No icon^d^	1.0		1.0	
**5 (5 icons)**		4.1	2.8–5.9***	3.1	2.2–4.5***
	No icon^d^	1.0		1.0	

^a ^Icons are as follows: S = stethoscope, O = otoscope, RH = reflex hammer, SS = surgical scrubs,*P* = pen.

^b ^Odds ratio.

^c ^Confidence interval.

^d ^Photo with no icon group was the reference group for each of the models.

^e ^95% CI was rounded to 1.00 but the *P *value was >.05.

*P < .05, **P < .01, ***P < .001.

## Discussion

When only 1 icon was presented in the photograph, the stethoscope was associated with the highest odds for the person being considered honest, trustworthy, moral, honorable, ethical, or genuine, or a combination of these. The stethoscope evoked strong positive perceptions. There were no differences in scores on any characteristics for any combination of 2 icons. However, photographs with 3 or more icons had significantly higher scores for all characteristics than photographs with 0 or 1 icon. Literature on the impact of prominently displaying icons in medical consulting rooms concluded that they may determine the extent to which a doctor is perceived to be open to the ideas, concerns, and expectations of the patient in the consultation [16]. Similarly, our data suggest that items of medical equipment may influence the perceptions patients have of a person in a photograph.

### Strengths and Limitations

The same images, albeit a limited random selection, were shown to all respondents. This is a possible limitation because the demand characteristics placed on the participants may have been enough to explain the increasing trustworthiness ratings with increasing number of medical devices in the photographs. The participants may have worked out that their ratings were supposed to be based on the changing elements of the pictures and that more devices should indicate greater trustworthiness. However, the relative trustworthiness ratings of types of devices might be free from demand characteristics, and we were able to estimate this from the data. The demographic characteristics of the respondents were similar to those of patients who generally visit general practitioners in Australia, and most were women over 45 years of age [20]. The data suggest that including certain icons in the static image of medical practitioners can improve the patient’s perception of the doctor’s trustworthiness. On the other hand, there were a number of limitations. We assumed everyone would recognize all items of medical equipment and the surgical scrubs. This could not be confirmed and informal feedback to the researchers conducting the interviews suggested that some of the respondents did not recognize the surgical scrubs or the reflex hammer in particular. Some items of medical equipment are no longer used exclusively by doctors and are now also commonly used by other health care professionals including nurses, physiotherapists, and paramedical staff. The data may not be generalizable because respondents were mostly female, with none aged younger than 18 years. All participants, or their relatives, had consulted a doctor recently and all were attending a community pharmacy. There were other signs in this study that we did not take into consideration and that respondents would have interpreted through community-accepted codes. These included facial expression, color of the person’s shirt, his race (white European), and his gender. These signs would have been likely to affect respondents’ assessment of trustworthiness. In this study, we did not test the icons with video footage—the pictures were static images and not animated as they would appear in an online consultation with a doctor. Finally, we also acknowledge the importance of context (which both patient and professional will bring to the consultation) with additional cues (identifying the professional as a doctor), which may either enhance or diminish the effect of the icon.

### Future Research

In this study we introduced participants to a series of photographs of a person along with a variety of items associated with doctors. These items were used because they are commonly recognized symbols or icons of medical practice that patients use to make meaning of a medical consultation. In Peircean semiotics, each of the items we employed is more properly an index because the item is connected directly in some way to the functions we ascribe to doctors, and hence the signifier is not entirely arbitrary [21]. It could be argued that other symbols, not usually seen in medical practice, may also be associated with trustworthiness, but that does not necessarily detract from the finding that a perception of trustworthiness is correlated with the presence of certain items used in medical consultations. It is possible that this study has further established what is already known: stethoscopes in particular are associated with medicine, and doctors are considered trustworthy. At the least, however, this study demonstrates additionally that the presence of certain icons is strongly correlated with perceived trustworthiness. The study invites the further question of whether the presence of such icons can be a way to more quickly build a relationship of trust, which is considered an important element in therapeutic relationships [22]. In future studies for consultations in which the doctor and patient are not in the same room, we need to demonstrate that even if the equipment is not deployed, displaying this equipment on-screen can have a measurable impact on outcomes for patients. Similarly, the impact of medical equipment may also apply in circumstances in which doctors consult patients in person. This too needs to be further explored.

### Conclusions

When doctors appear in static images, 3 or more icons of medical equipment may be helpfully included in the images, one of which should be a stethoscope. These icons are likely to have a positive influence on patients’ perceptions of the trustworthiness of the practitioner.

## Multimedia Appendix 1

No icon.

## Multimedia Appendix 2

Otoscope.

## Multimedia Appendix 3

Reflex hammer.

## Multimedia Appendix 4

Pen.

## Multimedia Appendix 5

Stethoscope.

## Multimedia Appendix 6

Surgical scrubs.

## Abbreviations

CI: confidence interval
OR: odds ratio
SCS: Source Credibility Scale
