# Supplementary figures and images for: Impact of the Presence of Medical Equipment in Images on Viewers’ Perceptions of the Trustworthiness of an Individual On-Screen
Source: J Med Internet Res. 2012 Jul 10;14(4):e100. doi: 10.2196/jmir.1986 (PMC3409609; doi:10.2196/jmir.1986)

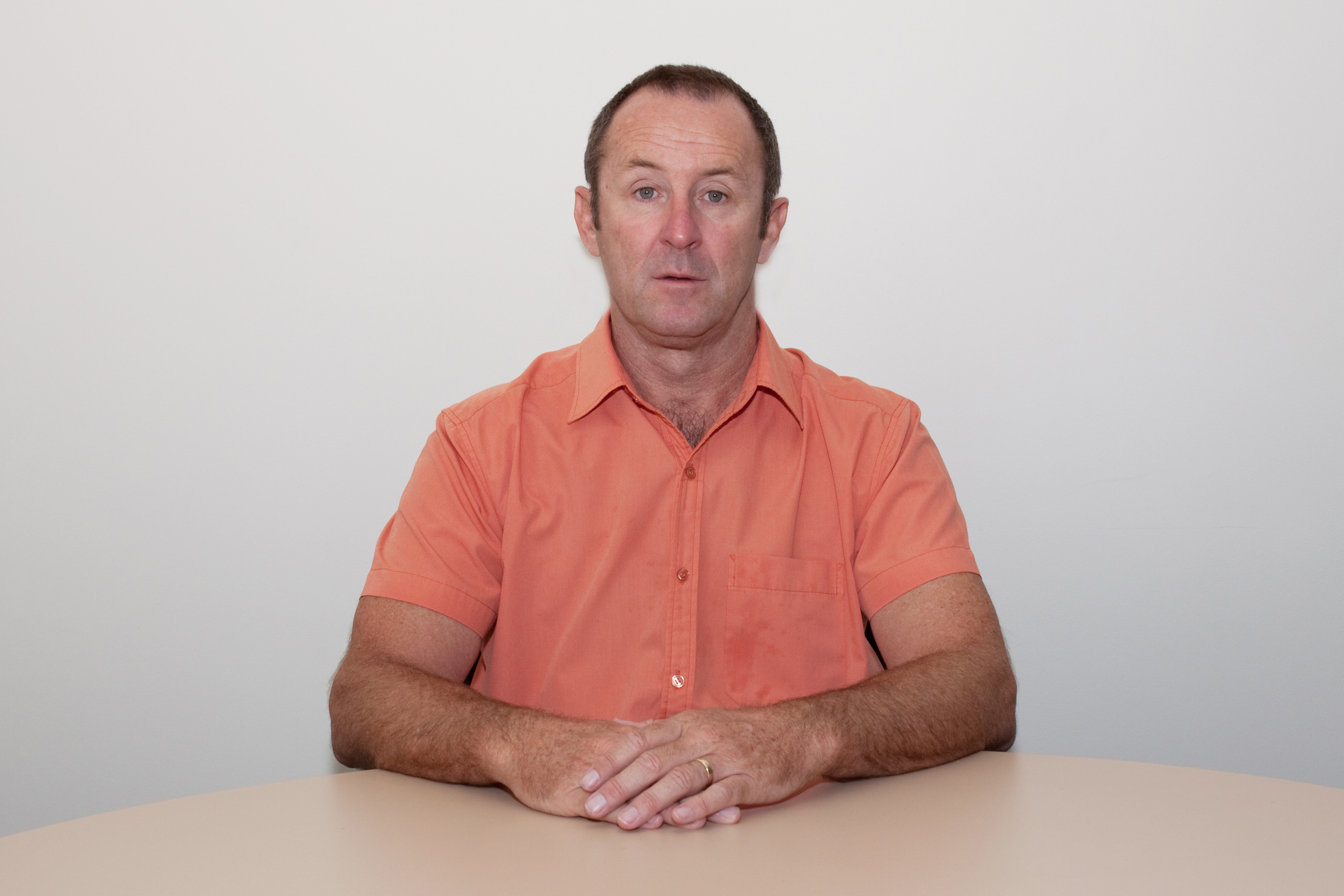

Supplement: Supplementary file 1 [file jmir_v14i4e100_app1.jpg]

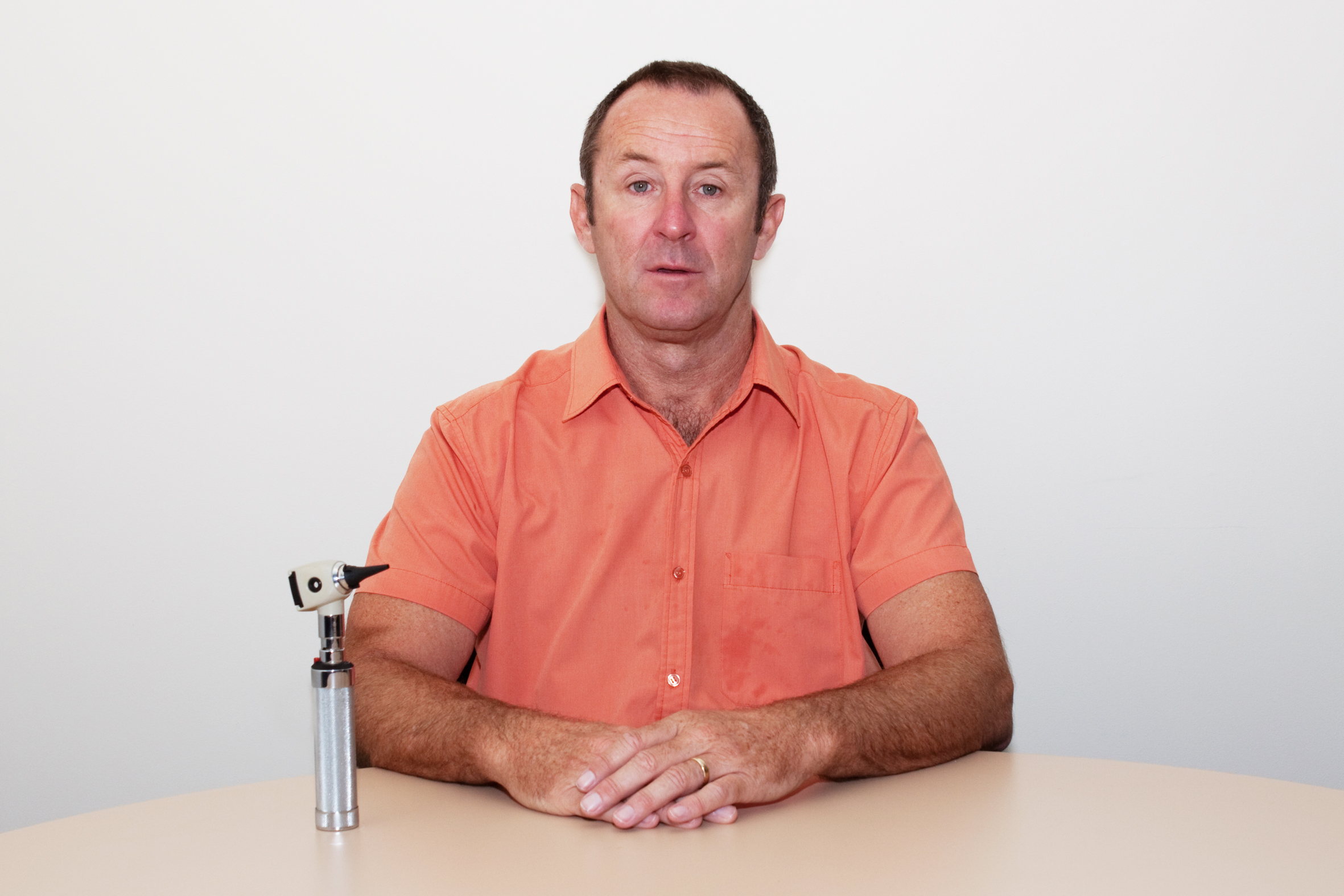

Supplement: Supplementary file 2 [file jmir_v14i4e100_app2.jpg]

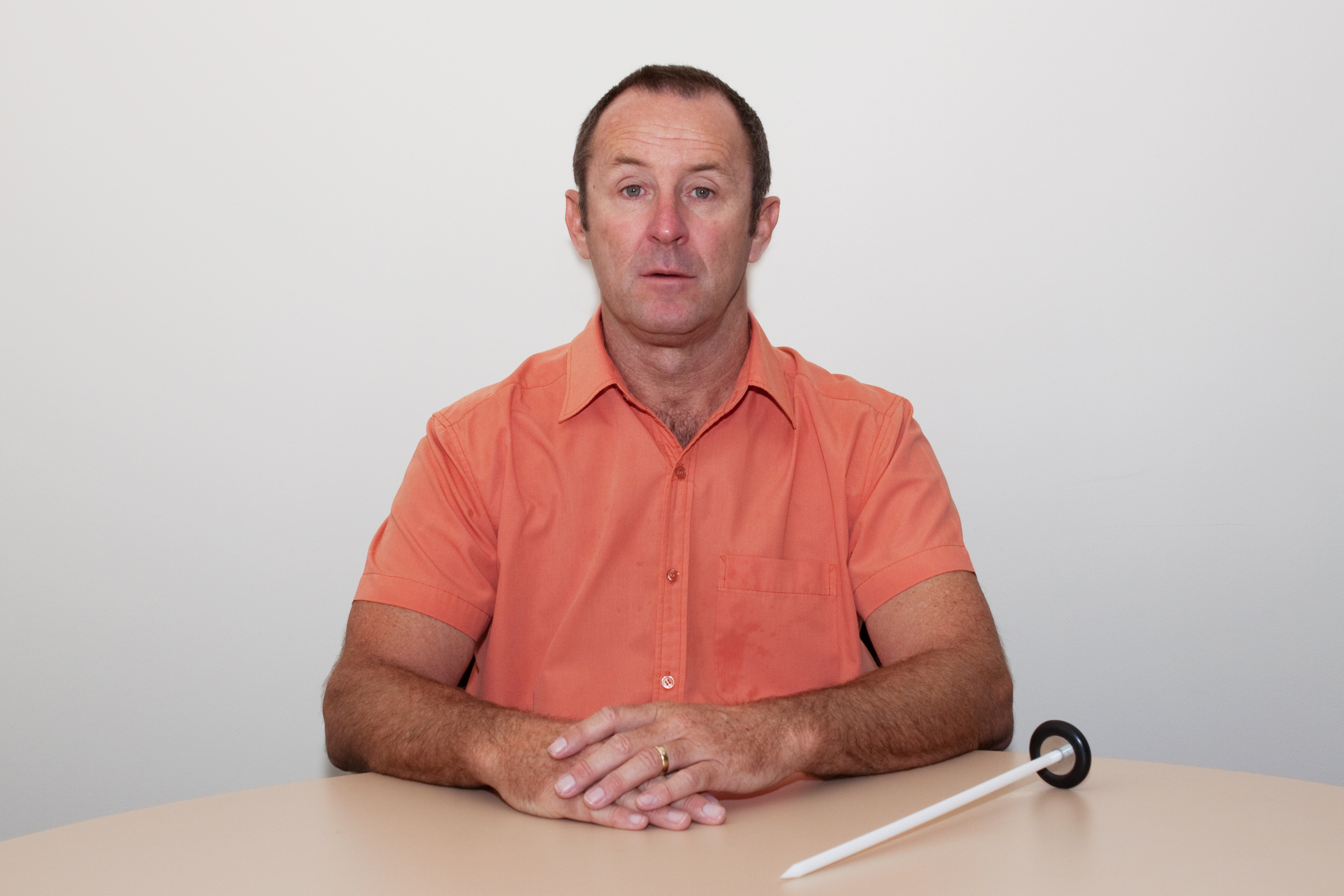

Supplement: Supplementary file 3 [file jmir_v14i4e100_app3.jpg]

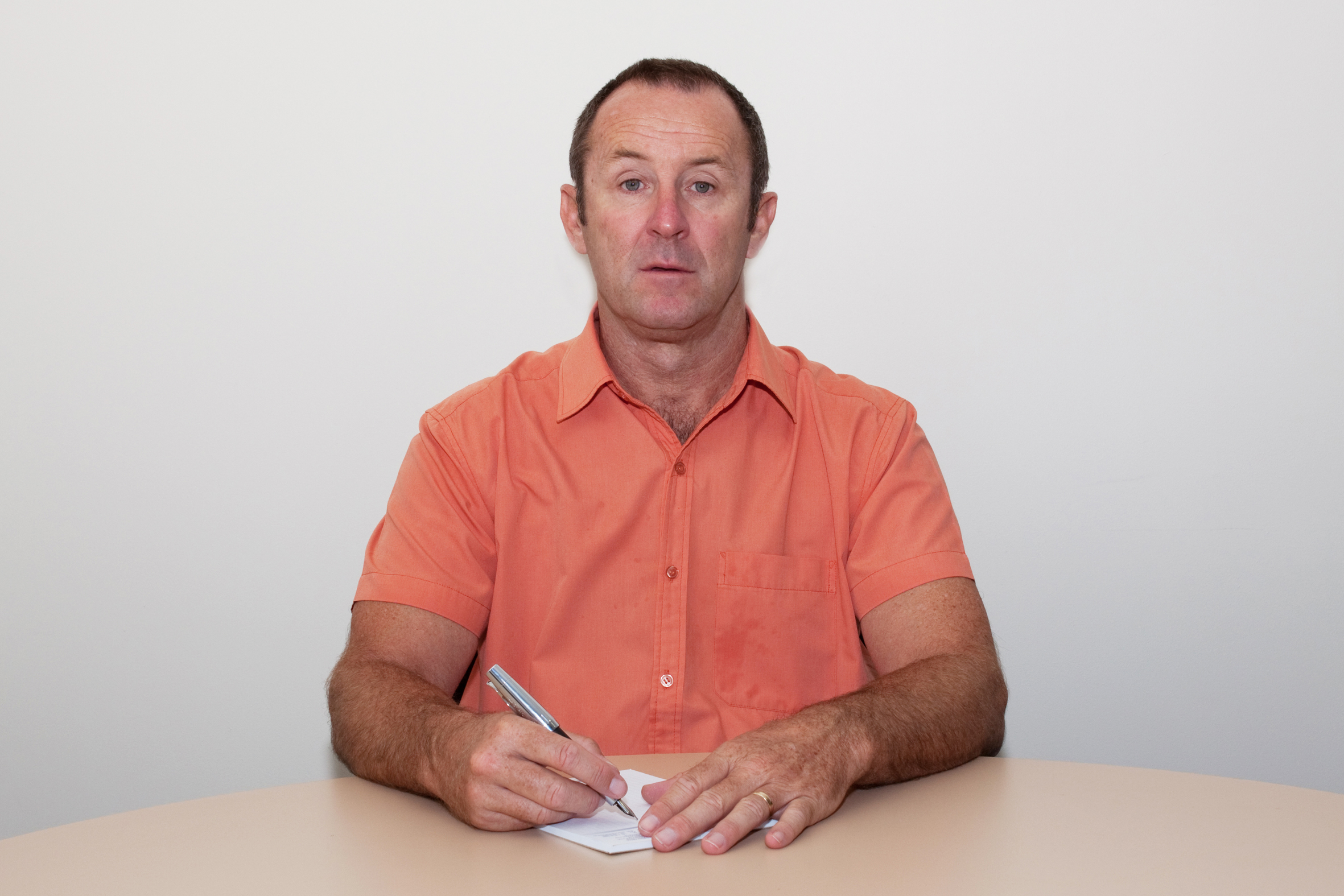

Supplement: Supplementary file 4 [file jmir_v14i4e100_app4.jpg]

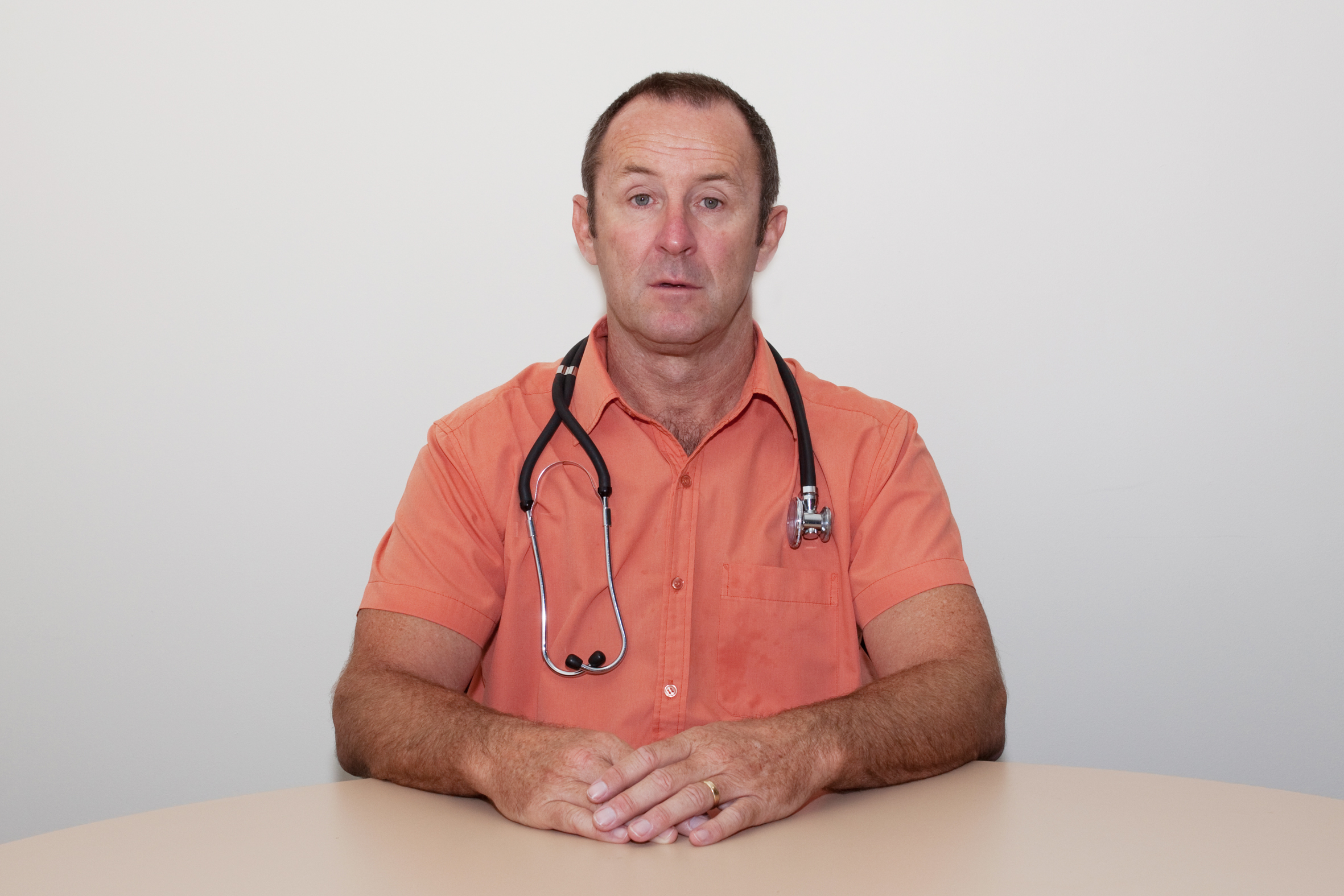

Supplement: Supplementary file 5 [file jmir_v14i4e100_app5.jpg]

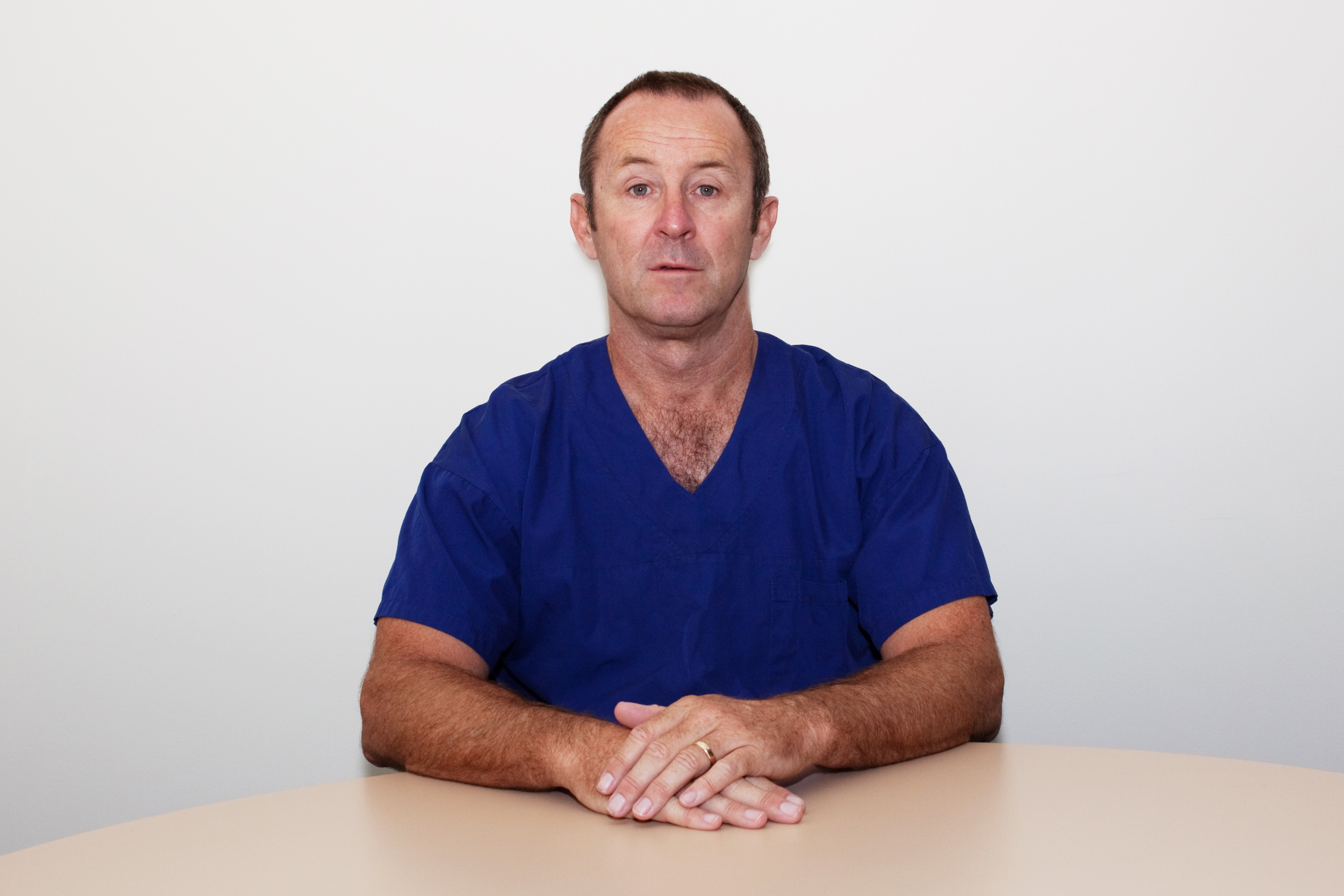

Supplement: Supplementary file 6 [file jmir_v14i4e100_app6.jpg]
